# Comparative proteinuria management of different angiotensin-converting enzyme inhibitors or angiotensin receptor blockers for normotensive patients with CKD: a Bayesian network meta-analysis

**DOI:** 10.7717/peerj.8575

**Published:** 2020-03-12

**Authors:** Huizhen Ye, Zhihao Huo, Peiyi Ye, Guanqing Xiao, Zhe Zhang, Chao Xie, Yaozhong Kong

**Affiliations:** Nephrology Department, The First People’s Foshan Hospital, Foshan, Guangdong, China

**Keywords:** Proteinuria reduction, Normotension, Chronic kidney disease, ACEI, ARB, Bayesian network analysis.

## Abstract

**Background:**

Both angiotensin-converting enzyme inhibitors (ACEIs) and angiotensin receptor blockers (ARBs) are blood pressure-lowering agents, but they are also being used to control proteinuria in early chronic kidney disease (CKD) patients. However, clinically, some patients present merely proteinuria without hypertension. No guidelines pointed out how to select treatments for proteinuria in normotensive patients. Thus, we conducted a Bayesian network analysis to evaluate the relative effects of different kinds of ACEI or ARB or their combination on proteinuria and blood pressure reduction.

**Methods:**

The protocol was registered in PROSPERO with ID CRD42017073721. A comprehensive literature database query was carried out systematically according to PICOS strategies. The primary outcome was reduction in proteinuria, and the secondary outcomes were eGFR reduction and blood pressure reduction. Random-effects pairwise and Bayesian network meta-analyses were used to estimate the effect of different regimens.

**Results:**

A total of 14 RCTs with 1,098 patients were included in the analysis. All treatment strategies of ACEI, ARB or their combination had significantly greater efficacy in reducing proteinuria than placebo in normotensive CKD patients. The combination therapy of olmesartan+temocapril had the highest probability (22%) of being the most effective treatment to reduce proteinuria in normotensive CKD patients. Olmesartan and lisinopril ranked second (12%), and temocapril ranked third (15%) but reduced blood pressure less than placebo. For IgA nephropathy, the combination therapy of olmesartan+temocapril also had the highest probability (43%) of being the best antiproteinuric treatment, while enalapril had the highest probability (58%) of being the best antiproteinuric therapy for diabetic nephropathy.

**Conclusions:**

The combination therapy of olmesartan plus temocapril appeared to be the most efficacious for reducing proteinuria in normotensive CKD patients and IgA nephropathy, but the clinical application should be balanced against potential harms. Temocapril can be an option when practitioners are searching for more proteinuria reduction but less blood pressure variation. In normotensive diabetic nephropathy, monotherapy with the ACEI enalapril seems to be the most efficacious intervention for reducing albuminuria. Future studies are required to give a more definitive recommendation.

## Introduction

Chronic kidney disease (CKD) has become a significant public health problem. The National Center for Chronic Disease Prevention and Health Promotion reported a 15% overall prevalence of CKD in adults in the United States, suggesting there are approximately 37 million people with CKD in 2019 (National Center for Chronic Disease Prevention and Health Promotion, 2019). Proteinuria is one of the most common signs for early CKD patients. Promoting inflammation and fibrosis of kidneys, proteinuria has been perceived as a strong marker of kidney damage, which is closely related to a high risk of CKD progression (Avasare & Radhakrishnan, 2015; Chen, Wada & Chiang, 2017). Angiotensin receptor-blockers (ARBs) and angiotensin-converting enzyme inhibitors (ACEIs) are two kinds of blood pressure-lowering agents that are also used to control proteinuria in early CKD patients on the basis of clinical practice guidelines.

Indeed, some patients present merely proteinuria without hypertension clinically, especially among the early CKD patients. A previous meta-analysis (Geng et al., 2014) suggested that compared with the placebo group, the ARB group had a significant reduction in urinary protein excretion and better renoprotective effects in normotensive patients with CKD, but it did decrease both diastolic and systolic blood pressure. The reduction in blood pressure in normotensive patients may sometimes result in hypotension. What should be recommended for more proteinuria reduction and less blood pressure reduction in normotensive patients with CKD? A recent network meta-analysis by Huang et al. (2017) reported that the ACEI-ARB combination therapy of trandolapril+candesartan was the most efficacious in reducing albuminuria for normotensive diabetic patients. The study only included the diabetic patients, which means that the results cannot be generalized to normotensive patients with other kinds of CKD. It also did not report the effects on blood pressure reduction, which is important for clinical practitioners.

The objective of this article is to evaluate the relative effects of different kinds of ACEI or ARB or their combination on proteinuria reduction, including which therapy would be more suitable for normotensive patients with proteinuria but who need less blood pressure fluctuation.

Bayesian network analysis is an extension of traditional meta-analysis, which can make indirect comparisons of two treatments through a common comparator in the absence of head-to-head direct randomized controlled trials (Herrera-Gomez et al., 2019). Therefore, we performed a Bayesian network analysis to evaluate the relative proteinuria reduction and blood pressure changes by various ACEIs or ARBs or their combination for normotensive patients with CKD.

## Methods

### Study design

Our network meta-analysis was conducted by the rules of Preferred reporting items for systematic review and meta-analysis protocols (PRISMA-P) 2015 (Shamseer et al., 2015) and the PRISMA Extension Statement for Reporting of Systematic Reviews Incorporating Network Meta-analyses of Health Care Interventions (Hutton et al., 2015). This study was performed using the Bayesian network analysis model, which is based on generalized linear models . The protocol of this study was registered in https://www.crd.york.ac.uk/prospero/, an international register of systematic reviews, which can be available with ID CRD42017073721.

### Search strategy

We searched PubMed, the Cochrane Liberary, Embase, CBMdisc, Wanfang database, and CNKI with the PICOS strategy with advanced searches and Mesh searches with a cut-off date of June 2019 without language restrictions. We also searched the System for Information on Grey literature (SIGLE), master’s and doctoral dissertations, and meeting records in the Chinese database CNKI for grey literature. The text words for searching included “normotensive, proteinuria, albuminuria, microalbuminuria, angiotensinreceptor-blockers, ARB, angiotensin-converting enzyme inhibitor, ACEI, the names of currently available ARBs or ACEI (losartan, valsartan, irbesartan,candesartan, telmisartan, eprosartan, olmesartan, imidapril, enalapril, lisinopril, captopril, cilazapril, ramipril, perindopril, and fosinopril)”. Meanwhile, we also checked the reference lists of review articles, meta-analysis, and original studies in order to cover more eligible trials.

The PICOS for our review was as follows:

**Intervention:** angiotensin-receptor-blockers, ARB, angiotensin-converting enzyme inhibitor, ACEI, the names of currently available ARBs or ACEIs (losartan, valsartan, irbesartan, candesartan, telmisartan, eprosartan, olmesartan, imidapril, enalapril, lisinopril, captopril, cilazapril, ramipril, perindopril, and fosinopril).

**Comparator:** angiotensin-receptor-blockers, ARB, angiotensin-converting enzyme inhibitor, ACEI, placebo, other antihypertensive agents

**Outcomes**

**Primary outcomes:** urinary protein excretion: proteinuria, urinary albumin excretion

**Secondary outcomes:**

Glomerular filtration rate (GFR).

Blood pressure (BP), systolic blood pressure (SBP)

**Study design**: randomized controlled trials (RCTs)

### Inclusion and exclusion criteria

Studies meeting the following inclusive criterion were eligible: (1) randomized controlled trials (RCTs); (2) participants aged 18 years or older; (3) with kidney disease and reporting proteinuria; (4) specifically analyzing and stating normotension of all the participants. Patients with dialysis or kidney transplantation were excluded.

### Data extraction and quality assessment

Data was extracted from all primary studies by two independent researchers (Huizhen Ye and Zhihao Huo) according to the registered protocol, including article information about CASP Checklist (Critical Appraisal Skills Programme, 2018) for quality assessment, first author’s name, publication year, geographic region and participant characteristics (sample size, mean age, gender, duration of intervention). We resolved disagreements through discussion with a third researcher to reach consensus (Yaozhong Kong).

In addition, we used the five-point Jadad Score to assess the methodological quality of studies, which mainly evaluated three aspects (randomisation, blinding, withdrawals and dropouts) of all the studies. Score ≤2 points was defined as low quality, while score ≥3 points was ranked as high quality. We also used the CASP Checklist, an 11-question list, to help us make sense of the RCTs we included and complete the quality assessment. It is made up three sections concentrating on three problems: (A) Are the results of the study valid? (B) What are the results? (C) Will the results help locally? Only for studies with more than two “Y” answers in section (A) is worth proceeding with the remaining questions.

### Data analysis

The primary outcome in this study was proteinuria reduction. The secondary outcomes were the reduction in blood pressure and GFR. A standard pairwise meta-analysis of the same interventions was performed using ADDIS 1.16.5 software (Aggregate Data Drug Information System, The Netherlands) with a random-effects model. Heterogeneity was assessed with the I^2^ metric, and Bayesian network analysis was conducted using ADDIS 1.16.5 software in a Bayesian Markov chain Monte Carlo framework with a consistency model or an inconsistency model. For the ranking of the interventions, stochastic multi-criteria acceptability analysis (SMAA)-based models were used (Van Valkenhoef et al., 2013). For antiproteinuric analysis, 4 chains including 20,000 burn-ins, 50,000 simulation iterations, 10,000 inference samples and a thinning interval of 10 for each chain were applied. Convergence was assessed by comparing within-chain and between-chain variance to calculate the potential scale reduction factor (PSRF) (Jing et al., 2012).The PSRF was extremely close to 1.00, showing good convergences of iterations.

The Consistency and inconsistency of the RCTs included in the network was also assessed by the Higgins model with Stata MP 14 (64-bit) (Computer Resource Center, USA), inconsistency factor and node-splitting analysis with ADDIS 1.16.5 software, to explore whether the direct and indirect evidence were in agreement. Conclusions could be drawn by a consistency model if no relevant inconsistency existed.

A basic network diagram was drawn with Stata MP 14 (64-bit) to show the connections between all the included treatments.

## Results

**Figure 1:**
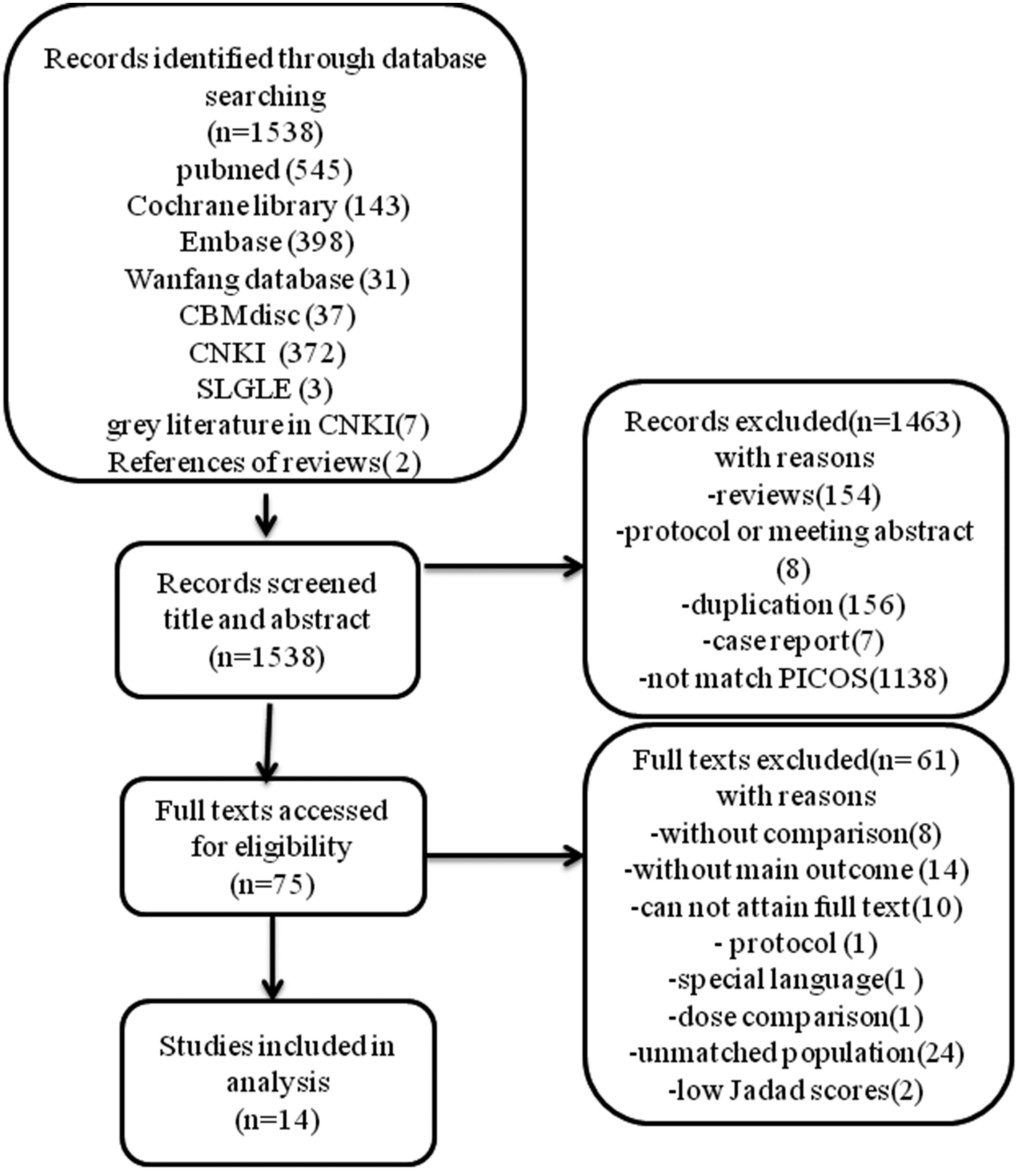
Flow diagram.

Literature selection and study characteristics. A total of 1,538 records were identified from our initial literature query. After title and abstract screening, a total of 75 records remained and were assessed for full texts screening. Since 10 full texts could not be obtained, 65 articles were read through, and articles were excluded for various reasons listed in the flowchart (Fig. 1). Finally, 16 RCT studies met the inclusion and exclusion criteria, but 2 studies were excluded after quality assessment for low Jaded score (1 point), so 14 RCTs with 1098 patients were included in the meta-analysis. The flowchart summarizing the study selection process is provided in Fig. 1. A summary of the characteristics of the included studies is shown in Table 1 and CASP checklist of included studies presented in Table 2.

**Table 1:** Characteristics of included RCTs.

**Reference**	**Country of origin**	**Jadad scores**	**Number for interventions**	**Interventions**	**Age**	**Sex (Male/ Female)**	**N**	**Follow-up (months)**	**Nephropathy**
Horita et al. (2004)	Japan	2	3	G1:temocapril 1 mg qd G2:losartan 12.5 mg qd G3: temocapril 1 mg+losartan 12.5 mg qd	G1:39.6 ± 10.8 G2:42.7 ± 12.0 G3:39.6 ± 10.4	G1:4/6 G2: 5/5 G3:5/6	31	6	IgA Nephropathy
Maschio et al. (1994)	Italy	4	2	G1:fosinopril 20 mg qd G2: placebo	NS	NS	78	8	IgA Nephropathy
Nakamura et al. (2007)	Japan	3	3	G1:olmesartan 10 mg qd G2:temocapril 2 mg qd G3:olmesartan 10 mg+ temocapril 2 mg qd	G1:34 ± 7 G2:31 ± 8 G3:31 ± 7	G1:5/3 G2:4/4 G3:4/4	24	3	IgA Nephropathy
Odabas et al. (2001)	Turkey	2	2	G1:losartan 50 mg qd G2:placebo	G1:34.3 ± 5.9 G2:32.2 ± 4.3	G1:14/8 G2:14/8	44	24	Secondary Amyloidosis
Ravid et al. (1993)	Israel	5	2	G1:enalapril 10 mg qd G2:placebo	G1:43.5 ± 3 G2:44.8 ± 3.5	G1:21/28 G2:21/24	94	60	Diabetic Nephropathy
Renke et al. (2004)	Poland	2	3	G1:losartan 25 mg qd G2: enalapril 10 mg qd G3::losartan 25 mg+enalapril 10 mg qd	G1:40.4 ± 11.9 G2:43.4 ± 10.1 G3:37.7 ± 12.7	G1:7/11 G2:12/6 G3:11/5	52	9	IgA Nephropathy
Shen et al. (2012)	China	3	2	G1:losartan 50 mg qd G2:placebo	G1:50.2 ± 10.4 G2:49.1 ± 11.5	G1:58/54 G2:56/58	226	12	Nondiabetic Chronic Kidney Disease
Shimizu et al. (2008)	Japan	3	2	G1:losartan 12.5 mg qd G2:placebo	G1:36.0 ± 8.5 G2:35.7 ± 8.1	G1:11/7 G2:6/12	36	12	IgA Nephropathy
Kosmadakis et al. (2010)	Greece	2	2	G1:lisinopril 10 mg qd G2:losartan 100 mg qd	G1:52.1 ± 15.3 G2:50.5 ± 15.5	G1:6/7 G2:7/7	27	12	idiopathic membranous nephropathy
Acbay (2001)	Turkey	2	2	G1:enalapril 5 mg qd G2:losartan 25 mg qd	Total:29.4 ± 5.2	NS	16	2	Diabetic Nephropathy
Usta et al. (2003)	Turkey	3	2	G1:losartan 50 mg qd G2:placebo	G1:32 ± 10 G2:32 ± 13	G1:8/5 G2:6/4	23	12	Focal Segmental Glomerular Sclerosis
Agha et al. (2009)	Pakistan	3	2	G1:losartan 50 mg qd G2:placebo	G1:53.9 ± 11.1 G2:54.7 ± 10.9	NS	361	6	Diabetic Nephropathy
Atmaca & Gedik (2006)	Turkey	2	3	G1: lisinopril 10 mg qd G2:losartan 50 mg qd G3:lisinopril 10 mg+losartan 50 mg qd	G1:55.1 ± 8.9 G2:55.1 ± 9.2 G3:55.1 ± 9.6	G1:4/5 G2:4/5 G3:3/5	26	12	Diabetic Nephropathy
Nakamura et al. (2002)	Japan	3	4	G1:trandolapril 2 mg qd G2:candesartan 8 mg qd G3:trandolapril 2 mg+candesartan 8 mg qd	G1:57.0 ± 9.5 G2:56.5 ± 10.0 G3:57.8 ± 9.0 G4:54.8 ± 9.3	G:10/5 G2:11/4 G3:10/5	60	18	Diabetic Nephropathy

**Notes.**

NS not state G1 Group 1 G2 Group 2 G3 Group 3 G4 Group 4

Values are mean [SD].

**Table 2:** CASP checklist of included RCTs.

**Reference**	**Section A**						**Section B**			**Section C**		
	**Q1**	**Q2**	**Q3**	**Q4**	**Q5**	**Q6**	**Q7 (1. What outcomes were measured ?)**	**(2.Is the primary outcome clearly specified ?)**	**Q8: confidence limits metioned?**	**Q9**	**Q10**	**Q11**
Horita et al. (2004)	Y	Y	Y	Y	Y	Y	proteinuria, SBP, DBP, MAP, serum creatinine, serum total protein	Y	NS	Y	Y	Y
Maschio et al. (1994)	Y	Y	Y	Y	NS	Y	proteinuria, eGFR, MAP	Y	NS	Y	Y	Y
Maschio et al. (1994)	Y	Y	Y	N	Y	Y	proteinuria, eGFR, serum creatinine, L-FABP,8-OHdG	Y	NS	Y	Y	Y
Odabas et al. (2001)	Y	NS	Y	NS	Y	Y	proteinuria, serum creatinine	Y	NS	Y	Y	Y
Ravid et al. (1993)	Y	Y	Y	Y	Y	Y	Serum creatinine, Proteinuria, MBP	Y	Y	Y	Y	Y
Renke et al. (2004)	Y	Y	Y	N	Y	Y	proteinuria, SBP, DBP, serum creatinine	Y	NS	Y	Y	Y
Shen et al. (2012)	Y	Y	Y	N	Y	Y	proteinuria, eGFR, SBP, DBP, serum creatinine, serum uric acid	Y	NS	Y	Y	Y
Shimizu et al. (2008)	Y	Y	Y	N	Y	Y	proteinuria, serum uric acid, eGFR and so on	Y	NS	Y	Y	Y
Kosmadakis et al. (2010)	Y	Y	Y	NS	Y	Y	proteinuria, GFR, MAP and so on	Y	NS	Y	Y	Y
Acbay (2001)	Y	Y	Y	Y	Y	Y	SBP, DBP, UAER	Y	Y	Y	Y	Y
Usta et al. (2003)	Y	Y	Y	Y	Y	Y	MAP, proteinuria, creatinine and so on	Y	NS	Y	Y	Y
Agha et al. (2009)	Y	Y	Y	Y	Y	Y	SBP, DBP, serum creatinine, 24-hour urinary microalbumin	Y	NS	Y	Y	Y
Atmaca & Gedik (2006)	Y	Y	Y	Y	Y	Y	24-hour UAER	Y	NS	Y	Y	Y
Nakamura et al. (2002)	Y	Y	Y	NS	Y	Y	UAE, BP	Y	Y	Y	Y	Y

**Notes.**

UAER urinary albumin excretion SBP systolic blood pressure DBP diastolic blood pressure MAP mean arterial pressure; L-FABP Liver-type fatty acid-binding protein

Q1: Did the trial address a clearly focused issue? Q2: Was the assignment of patients to treatments randomised? Q3: Were all of the patients who entered the trial properly accounted for at its conclusion? Q4: Were patients, health workers and study personnel ‘blind’ to treatment? Q5: Were the groups similar at the start of the trial? Q6: Aside from the experimental intervention, were the groups treated equally? Q7: How large was the treatment effect? Q8: How precise was the estimate of the treatment effect? Q9: Can the results be applied to the local population, or in your context? Q10: Were all clinically important outcomes considered? Q11: Are the benefits worth the harms and costs?

**Figure 2:**
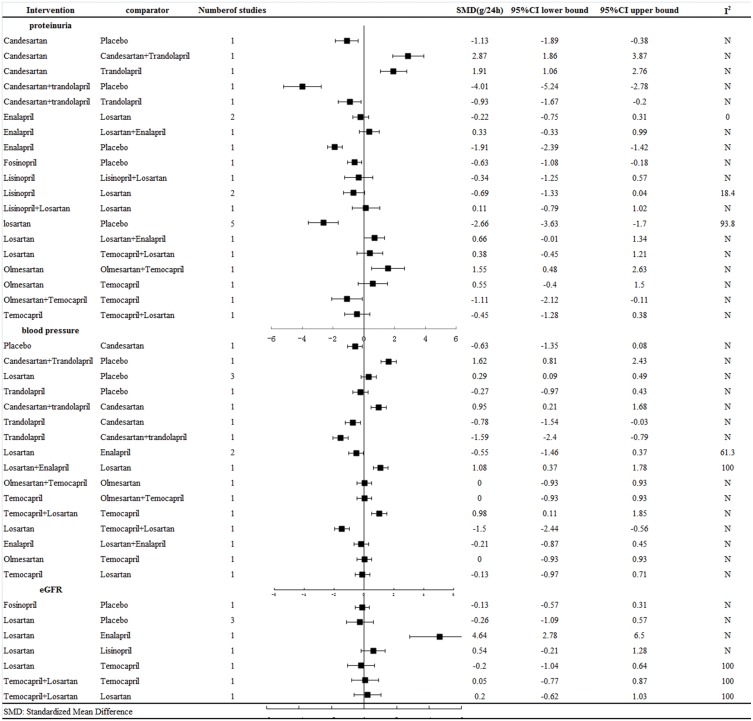
Response rates for efficacy in meta-analyses of direct comparisons between each pair of drugs.

Results of meta-analysis. Figure 2 shows the meta-analysis results for proteinuria, blood pressure and GFR reduction among the normotensive CKD patients, revealing that: (1) all the treatment strategies had a significantly better effect on reducing proteinuria than placebo; (2) the combination therapy of candesartan and trandolapril and the ACEI trandolapril alone were better than candesartan alone, with SMD = 2.87 (95% CI [1.86–3.87]) and SMD = 1.91 (95% CI [1.06–2.76]), respectively; (3) trandolapril alone, with SMD = −0.93 (95% CI [−1.67 to −0.2]), was not better than combination treatment with candesartan plus trandolapril ; (4) combination therapy with olmesartan and temocapril, with SMD = 1.55 (95% CI [0.48–2.63]), was better than monotherapy of olmesartan.

Results of Bayesian network analysis.

**Figure 3:**
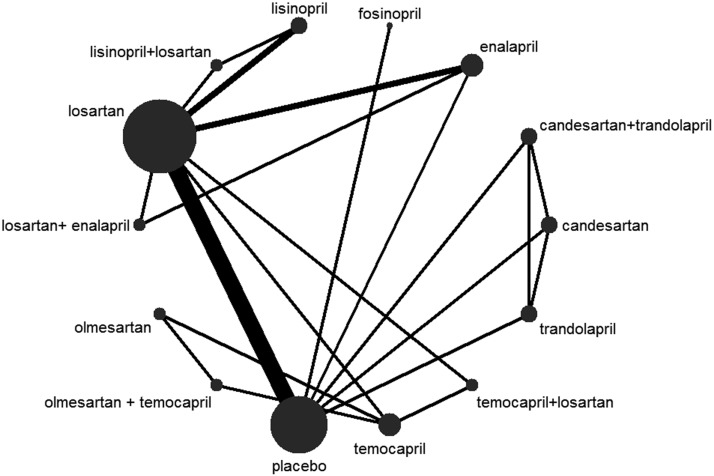
Network plot.

First, proteinuria reduction was reported in all 14 trials(Acbay, 2001; Agha et al., 2009; Atmaca & Gedik, 2006; Horita et al., 2004; Kosmadakis et al., 2010; Maschio et al., 1994; Nakamura et al., 2007; Nakamura et al., 2002; Odabas et al., 2001; Ravid et al., 1993; Renke et al., 2004; Shen et al., 2012; Shimizu et al., 2008; Usta et al., 2003). The network map of eligible comparisons for proteinuria reduction is presented in Fig. 3, showing the direct comparisons for our network analysis. The consistency was tested in three aspects: (1) The Higgins model calculated with Statas showed no evidence for inconsistency, with *P* = 0.877 (*P* > 0.05). (2) The inconsistency factor was −0.18 (95% CI [−8.84–6.86]). This 95% CI contained the neutral value (zero), suggesting the data was consistent. (3) Node-splitting analysis of proteinuria reduction, shown in Table 3, suggested the direct and indirect evidence on the split nodes were in agreement, with all the *P* > 0.05. Hence, we conducted the Bayesian network analysis with consistency random effect models (Table 4). Based on the Bayesian probability framework, the combination therapy of olmesartan+temocapril had the highest probability (22%) of being the most effective treatment to reduce proteinuria in normotensive CKD patients, followed by olmesartan (12%) and lisinopril (12%), with temocapril ranking third (15%) and placebo in tenth place.

**Table 3:** Node-splitting analysis of proteinuria reduction.

**Interventions**	**Direct effect**	**Indirect effect**	**Overall**	*P*-Value
Placebo vs. enalapril	0.20 (−4.17, 4.58)	1.62 (−2.15, 5.46)	0.99 (−1.51, 3.70)	0.56
Placebo vs. losartan	1.43 (−0.55, 3.46)	0.04 (−5.67, 5.57)	1.27 (−0.40, 2.97)	0.57
enalapril vs. losartan	−0.17 (−3.41, 2.96)	1.24 (−3.57, 6.01)	0.29 (−2.24, 2.66)	0.55
Values are mean [SD]				

**Table 4:** Outcomes of ranking from all RCTs.

	**Proteinuria reduction**	**BP reduction**	**eGFR reduction**
Placebo	10(16%)	7 (14%)	2(24%)
candesartan	13(14%)	/	/
candesartan+trandolapril	/	1 (37%)	/
enalapril	9(12%)	3 (20%)	7(86%)
fosinopril	14 (16%)	/	5(22%)
lisinopril	2(12%)	/	6(48%)
lisinopril+losartan	/	/	/
losartan	5(14%)	6(22%)	4(32%)
losartan+ enalapril	/	2(32%)	/
olmesartan	2(12%)	5(13%)	/
olmesartan + temocapril	1(22%)	/	/
temocapril	3(15%)	8(17%)	2 (24%)
temocapril+losartan	/	3 (14%)	1(35%)
trandolapril	/	11(51%)	/

**Notes.**

For Proteinuria reduction, rank 1 is best, rank N is worst.

For eGFR reduction, rank N is best, rank 1 is worst.

For BP reduction, rank N is best, rank 1 is worst.

Values are ranking number (probability).

Second, systolic blood pressure reduction was reported in 8 trials (Acbay, 2001; Agha et al., 2009; Atmaca & Gedik, 2006; Horita et al., 2004; Nakamura et al., 2002; Renke et al., 2004; Shen et al., 2012; Shimizu et al., 2008). As the rank was defined as a lesser blood pressure reduction being better, rank N reduces the blood pressure the least in the normotensive CKD patients. Via consistency random-effect models, trandolapril ranked in last place, while placebo ranked the seventh (14%), so trandolapril achieved less blood pressure reduction function than placebo.

Third, a total of 6 studies were included for network analysis of GFR reduction (Acbay, 2001; Horita et al., 2004; Kosmadakis et al., 2010; Shen et al., 2012; Shimizu et al., 2008; Usta et al., 2003). Rank N reduced GFR the least, suggesting the best renoprotective effect. The combination of temocapril plus losartan ranked first (35%), revealing that GFR was decreased the most, followed by placebo (24%).

Sensitivity analysis. We also performed sensitivity analysis that included IgA nephropathy and diabetic nephropathy respectively. For IgA nephropathy, olmesartan+temocapril had the highest probability (43%) of being the most effective treatment to reduce proteinuria in normotensive patients, followed by losartan+ enalapril (24%), temocapril ranking third (21%) and placebo in eighth place (21%). However, losartan+enalapril appeared to reduce the most blood pressure (44%), while placebo ranked eighth (39%). Regarding GFR reduction, temocapril+losartan still ranked first (43%), while placebo ranked fourth (39%).

For diabetic nephropathy, enalapril had the highest probability (58%) of being the most effective treatment to reduce proteinuria, followed by candesartan+trandolapril (40%), trandolapril ranking third (33%) and placebo in eighth place (45%).

## Discussion

Our Bayesian network analysis with 14 RCT studies and 1098 patients compared the relative effects of different kinds of ACEIs or ARBs or their combination in proteinuria reduction, blood pressure fluctuation and GFR reduction, seeking the best choice for normotensive patients with proteinuria. In our analysis, all the treatment strategies had a significantly better effect in reducing proteinuria than placebo in normotensive CKD patients. Our analysis also revealed that the combination therapy of olmesartan plus temocapril appeared to be most efficacious in reducing proteinuria in normotensive CKD patients. Temocapril reduced proteinuria significantly and led to less BP reduction than placebo, which seems to be a good option for patients with proteinuria and normotension. For normotensive IgA nephropathy, olmesartan+temocapril combination therapy seems to be the most efficacious therapy to reduce proteinuria. However, for diabetic nephropathy, the ACEI monotherapy of enalapril had the highest probability of being the best one to reduce proteinuria.

The results of the pairwise meta-analysis from this study were consistent with findings from previous meta-analyses. A 2012 meta-analysis comparing ACEI therapy with placebo showed a significant reduction in macroalbuminuria in diabetic patients without hypertension and a trend towards a benefit from the combination therapy of ACEI and ARB (Lv et al., 2012). Another, 2014 meta-analysis noted that ARBs have beneficial effects on reducing proteinuria in normotensive patients with renal disease(Geng et al., 2014). Our study also showed that all the treatments with ACEI, ARB or both lessened proteinuria significantly more than placebo in normotensive CKD patients, not merely in diabetic nephropathy patients.

Our analysis showed that the combination therapy of olmesartan plus temocapril appeared to be the most efficacious treatment for reducing proteinuria in normotensive CKD patients and IgA nephropathy patients. A previous meta-analysis (Cheng et al., 2012) also revealed that the combination of ACEI and ARB had a better antiproteinuric effect than monotherapy with ACEI or ARB. This result may be related to a larger glomerular capillary pressure reduction, more glomerular permselectivity improvement (Remuzzi et al., 1999; Woo et al., 2000) and inhibition of the secretion of some transforming growth factors, such as TGF-β1 (Scaglione et al., 2005; Song et al., 2003), when using combination therapy compared with monotherapy. A 2015 network meta-analysis (Palmer et al., 2015) investigated the side effects of the combination therapy of ACEI plus ARB. It showed a tendency towards acute kidney injury, stroke, hyperkalaemia, presyncope, and cough when compared to placebo, though the results were not significant. Fried’s study (Fried et al., 2013) in 2013 also mentioned that combination therapy increased the risk of hyperkalaemia and acute kidney injury. The 2012 KDIGO Clinical Practice Guideline for the Evaluation and Management of CKD (Inker et al., 2014) also did not recommend for combination therapy of ACEI plus ARB therapy for the obvious side effects mentioned above. Thus, the clinical application of combined ACEI and ARB should be balanced against the potential harm. Notably, Homma et al. (2004) reported that the proteinuria-reducing effect of combination therapy with ARB plus ACEI was independent of the effects on blood pressure. Thus, if we are searching for therapy with more proteinuria reduction and less blood pressure reduction, emocapril would be an option, as it ranked third in decreasing proteinuria and resulted in less blood pressure reduction than the placebo.

A previous network meta-analysis (Huang et al., 2017) found that the combination therapy of trandolapril plus candesartan was the best one to reduce albuminuria in normotensive diabetic patients. However, Mann et al. (2008) also mentioned that the combination therapy of ACEI and ARB reduced proteinuria to a greater extent but had less renal benefit in diabetic patients. Because of the increased harm of combination usage, the 2014 (Kidney Disease Outcomes Quality Initiative) KDOQI US commentary (Inker et al., 2014) advocated monotherapy with either ACEI or AEB. In our analysis, the monotherapy of enalapril had the highest probability of being the most effective treatment to reduce proteinuria, while the combination of trandolapril plus candesartan ranked second for normotensive diabetic patients. The difference may account for the various included criteria. However, for clinical practitioners, enalapril alone could be an option for normotensive diabetic patients.

There are several limitations to our analysis. First, we screened the records focusing on the primary outcome of proteinuria first, so that some of the studies reported no secondary endpoints. Second, measurements of proteinuria were not totally consistent across all included studies. For example, some studies reported albumin excretion rate (µg/min) but not 24-hour urinary protein excretion (g/d). Third, the conclusion cannot be applied to severe renal impairment or dialysis patients because most of the row data were from mild or no renal impairment patients. Finally, treatment dosing was not fixed during our analysis and some studies were even allowed drug dose titration.

## Conclusions

In summary, the combination therapy of olmesartan plus temocapril appears to have a stronger antiproteinuric effect for normotensive CKD patients and IgA nephropathy, but the clinical application should be balance against the potential harms. Temocapril might be an option when practitioners need to reduce proteinuria more but blood pressure less. For diabetic nephropathy, the ACEI monotherapy of enalapril had the highest probability of reducing albuminuria for normotensive diabetic patients. However, future studies are required to make a more definitive recommendation.

##  Supplemental Information

10.7717/peerj.8575/supp-1 Supplemental Information 1 Raw data

10.7717/peerj.8575/supp-2 Supplemental Information 2 PRISMA checklist

10.7717/peerj.8575/supp-3 Supplemental Information 3 Rationale and Contributions

## Additional Information and Declarations

### Data Availability

The following information was supplied regarding data availability: The raw measurements are available in the Supplementary Files.
