# Supplementary material for: Comparative proteinuria management of different angiotensin-converting enzyme inhibitors or angiotensin receptor blockers for normotensive patients with CKD: a Bayesian network meta-analysis
Source: PeerJ. 2020 Mar 12;8:e8575. doi: 10.7717/peerj.8575 (PMC7073241; doi:10.7717/peerj.8575)
Supplement: Supplemental Information 3 [file peerj-08-8575-s003.doc]

Supplemental Files:

1. The rationale for conducting the meta-analysis;

Response: The objective of this article is to evaluate the relative effects of different kinds of ACEI or ARB or their combination on proteinuria reduction, including which therapy would be more suitable for normotensive patients with proteinuria but who need less blood pressure fluctuation.However, the lack of original head-to-head direct trials laid barriers on this study.

Bayesian network analysis is an extension of traditional meta-analysis, which can make indirect comparisons of two treatments through a common comparator in the absence of head-to-head direct trials. Therefore, we performed a Bayesian network analysis to evaluate the relative proteinuria reduction and blood pressure changes by various ACEIs or ARBs or their combination for normotensive patients with CKD.

Network meta-analysis ,is an indirect treatment comparison technique(NMA), to compare two treatments in the situation where an indirect comparison between two treatments of interest can be obtained through more than one common comparator or linking treatment. For instance, consider a setting where there is interest in performing an indirect comparison between treatment A and treatment B. If trials have separately compared treatment A to C, treatment B to C, treatment A to D, and treatment B to D, this method allows investigators to incorporate results from trials in which the common comparator was C, as well as trials in which the common comparator was D. Thus, more than one common treatment can be used to conduct an indirect comparison between two treatments. NMA also allows determining the amount of agreement between the results obtained when different linking treatments are used. More details can be obtained in supplemental file named “rationale for network meta-analysis”.

2. The contribution that the meta-analysis makes to knowledge in light of previously published related reports, including other meta-analyses and systematic reviews.

Response: A previous meta-analysis(Geng et al. 2014) suggested that compared with the placebo group, the ARB group had a significant reduction in urinary protein excretion and better renoprotective effects in normotensive patients with CKD, but it did decrease both diastolic and systolic blood pressure. The reduction in blood pressure in normotensive patients may sometimes result in hypotension. What should be recommended for more proteinuria reduction and less blood pressure reduction in normotensive patients with CKD? A recent network meta-analysis by Huang R et al.(Huang et al. 2017) reported that the ACEI-ARB combination therapy of trandolapril+candesartan was the most efficacious in reducing albuminuria for normotensive diabetic patients. The study only included the diabetic patients, which means that the results cannot be generalized to normotensive patients with other kinds of CKD. It also did not report the effects on blood pressure reduction, which is important for clinical practitioners.

The objective of this study is to evaluate the relative effects of different kinds of ACEI or ARB or their combination on proteinuria reduction, including which therapy would be more suitable for normotensive patients with proteinuria but who need less blood pressure fluctuation.

Reference:

Geng DF, Sun WF, Yang L, En G, and Wang JF. 2014. Antiproteinuric effect of angiotensin receptor blockers in normotensive patients with proteinuria: a meta-analysis of randomized controlled trials. *J Renin Angiotensin Aldosterone Syst* 15:44-51. 10.1177/147032031247405

Huang R, Feng Y, Wang Y, Qin X, Melgiri ND, Sun Y, and Li X. 2017. Comparative Efficacy and Safety of Antihypertensive Agents for Adult Diabetic Patients with Microalbuminuric Kidney Disease: A Network Meta-Analysis. *PLoS One* 12:e0168582. 10.1371/journal.pone.0168582
